# Self-monitoring of health data by patients with a chronic disease: does disease controllability matter?

**DOI:** 10.1186/s12875-017-0615-3

**Published:** 2017-03-20

**Authors:** Martine W. J. Huygens, Ilse C. S. Swinkels, Judith D. de Jong, Monique J. W. M. Heijmans, Roland D. Friele, Onno C. P. van Schayck, Luc P. de Witte

**Affiliations:** 1 Department of Health Services Research, School for Public Health and Primary Care (CAPHRI), Maastricht University, P.O. Box 616, 6200 MD Maastricht, The Netherlands; 2 NIVEL, Netherlands Institute for Health Services Research, P.O. Box 1568, 3500 BN Utrecht, The Netherlands; 3 Tilburg School of Social and Behavioral Sciences, Tilburg University, Tranzo, P.O. Box 90153, 5000 LE Tilburg, The Netherlands; 4 Department of Family Medicine, School for Public Health and Primary Care (CAPHRI), Maastricht University, P.O. Box 616, 6200 MD Maastricht, The Netherlands; 5 Research Center Technology in Care, Zuyd University of Applied Sciences, P.O. Box 550, 6400 AN Heerlen, The Netherlands; 6 Centre for Assistive Technology and Connected Healthcare (CATCH), University of Sheffield, 217 Portobello, Sheffield, S1 4DP UK; 7 Centre for Care Technology Research, Maastricht, The Netherlands

**Keywords:** Self-monitoring, Chronic disease, Patients, Disease controllability, Self-management

## Abstract

**Background:**

There is a growing emphasis on self-monitoring applications that allow patients to measure their own physical health parameters. A prerequisite for achieving positive effects is patients’ willingness to self-monitor. The controllability of disease types, patients’ perceived self-efficacy and health problems could play an essential role in this. The purpose of this study is to investigate the relationship between patients’ willingness to self-monitor and a range of disease and patient specific variables including controllability of disease type, patients’ perceived self-efficacy and health problems.

**Methods:**

Data regarding 627 participants with 17 chronic somatic disease types from a Dutch panel of people with chronic diseases have been used for this cross-sectional study. Perceived self-efficacy was assessed using the general self-efficacy scale, perceived health problems using the Physical Health Composite Score (PCS). Participants indicated their willingness to self-monitor. An expert panel assessed for 17 chronic disease types the extent to which patients can independently keep their disease in control. Logistic regression analyses were conducted.

**Results:**

Patients’ willingness to self-monitor differs greatly among disease types: patients with diabetes (71.0%), asthma (59.6%) and hypertension (59.1%) were most willing to self-monitor. In contrast, patients with rheumatism (40.0%), migraine (41.2%) and other neurological disorders (42.9%) were less willing to self-monitor. It seems that there might be a relationship between disease controllability scores and patients’ willingness to self-monitor. No evidence is found of a relationship between general self-efficacy and PCS scores, and patients’ willingness to self-monitor.

**Conclusions:**

This study provides the first evidence that patients’ willingness to self-monitor might be associated with disease controllability. Further research should investigate this association more deeply and should focus on how disease controllability influences willingness to self-monitor. In addition, since willingness to self-monitor differed greatly among patient groups, it should be taken into account that not all patient groups are willing to self-monitor.

**Electronic supplementary material:**

The online version of this article (doi:10.1186/s12875-017-0615-3) contains supplementary material, which is available to authorized users.

## Background

In recent decades there has been a growing emphasis on self-monitoring applications in primary care. These applications allow patients to measure their own physical health parameters, such as blood pressure, blood glucose level and lung function [1–3]. Self-monitoring is a key aspect of patients’ self-management [4], especially in diseases like diabetes, asthma and heart failure. It offers the potential to create awareness of symptoms, bodily sensations, daily activities and cognitive processes and to provide information for action [5]. The effects of self-monitoring look promising: literature shows that it could improve self-management, symptom management and disease regulation, and could lead to reductions in complications, improved patients’ coping and attitudes toward their disease, realistic goal setting and an enhanced quality of life [6]. Self-monitoring is a broad term, including the monitoring of clinical parameters, symptom measures and daily activities [5]. In the current study we focus on individual self-monitoring of clinical parameters (such as: weight, blood pressure, blood glucose level and lung function) with the use of technical equipment.

With the introduction of new technologies, self-monitoring has become more convenient and accessible for patients. However, a prerequisite for achieving the positive effects of self-monitoring is the willingness of patients to self-monitor. Patients’ willingness to use technologies in health care is often studied with the Technology Acceptance Model (TAM) [7, 8]. This model theorizes that beliefs about perceived ease of use and perceived usefulness are the main constructs predicting user intention. A recent review study shows that the TAM is still the most important model used to identify the factors that influence the adoption of information technologies in health care [9]. This model had been extended and modified in recent years, such as in the Unified Theory of Acceptance and Use of Technology (UTAUT) [10]. Besides ease of use and perceived usefulness two other key constructs are included in the UTAUT model: social influence and facilitating conditions. However, we suggest that there are other underlying disease-specific and patient-specific factors that play an essential role in patients’ willingness to self-monitor.

For instance, the relevance of self-monitoring may not be the same for each disease type. For patients with diabetes and hypertension, for example, self-management goals are easy to define, such as optimizing blood glucose level and blood pressure, which are parameters that can easily be monitored by the patient. For patients with a disease like arthritis these goals are less concrete [11]. Moreover, other researchers suggest that in disease types such as diabetes, the feedback between action and change is rather direct and can clearly be observed by the patient, which can trigger the sensemaking process of performing self-management behaviour. For disease types such as cancer there are less direct and easily captured indicators that can activate this process [12].

Hence, it would seem that disease types differ in the extent to which they are controllable by the patients’ behaviour (e.g., using medication, nutrition and physical activity), which could be related to patients’ willingness to self-monitor. Some support for this was found in a recently performed focus group study [13]. In this study we found that patients with diabetes were more interested in the use of self-monitoring than patients with a Chronic Obstructive Pulmonary Disease (COPD) and a cardiovascular condition, because they mentioned that their own behaviour (nutrition, weight loss and medication) directly influenced their health, and that self-monitoring support could help them to influence their behaviour.

However, disease controllability does not only differ between disease types, but can also differ between individuals. Patients’ belief that they are capable of managing and controlling their disease is better known as self-efficacy [14]. This plays an important role in performing self-management behaviour [15] and might likewise influence patients’ willingness to self-monitor. Previous research found that higher perceived self-efficacy was associated with better blood glucose monitoring in patients with diabetes [16, 17].

Besides self-efficacy, the benefits that patients experience from self-monitoring might play an important role in their willingness to self-monitor. According to the Health Belief Model [18], perceiving higher benefits in relation to costs improves the performance of health behaviour. This is also found to be related to adherence to self-monitoring in patients with diabetes [19]. Experienced benefits regarding self-monitoring could be the reduction or prevention of disease symptoms. In our focus group study we found that patients with a chronic disease who experienced minimal health complaints were less willing to self-monitor because they expected fewer benefits. They did not expect improvements in their health, because their disease had little impact on their life, and were more focused on the perceived costs; the time it takes to do the self-monitoring [13]. Therefore in terms of self-monitoring we argue that patients who experience more severe health problems perceive higher benefits from self-monitoring (improvements in their health) in relation to the costs (doing the self-monitoring) and might likewise be more willing to self-monitor.

Up until now self-monitoring is often not yet integrated in standard care procedures. Moreover, the role of the patient and health care professional regarding the provision of self-monitoring is not yet defined. This study aimed to get more insight in willingness to self-monitor by patients with different chronic disease types. In this study our hypotheses which were based on the results of the focus group study are tested in a wider range of disease types to answer the following research question: what is the relationship between the controllability of disease types (disease specific) and patients’ perceived self-efficacy and health problems (patient specific) on the one hand, and patients’ willingness to self-monitor on the other. In addition, the influence of patients’ characteristics (gender, age, level of education and multimorbidity) on patients’ willingness to self-monitor will be investigated. Based on the previously performed focus group study we generated three hypotheses:

Disease-specific hypothesis:

1. The controllability of a certain type of chronic disease is related to patients’ willingness to self-monitor; patients with a chronic disease that can be, in general, properly kept under control by the patient will be more interested in self-monitoring than patients that have a disease that is less controllable by the patient.

Patient-specific hypotheses:

2. Patient’s perceived self-efficacy is related to their willingness to self-monitor; patients with high perceived self-efficacy are more interested in self-monitoring than patients that perceive low self-efficacy.

3. The severity of problems that patients experience with daily functioning is related to their willingness to self-monitor; patients that have moderate problems with daily functioning are more interested in self-monitoring than patients who perceive no problems with daily functioning. This holds to a certain extent; patients who experience many problems with daily functioning might not be able to do the monitoring anymore.

We investigated these hypotheses in a Dutch nationwide study of patients with the most prevalent chronic diseases.

## Methods

### Design and participants

Data from 1294 participants of the National Panel of people with Chronic illness or Disability (NPCD) were used for this cross-sectional study [20]. This panel was established by NIVEL (the Netherlands Institute for Health Services Research) and is a nationwide prospective panel study in the Netherlands. Participants with a chronic disease are recruited from random samples of general practices in the Netherlands. The following criteria were used for recruitment of the NPCD: being diagnosed with a somatic chronic disease (using the International Classification of Primary Care (ICPC)) by a certified medical doctor, being aged 15 or older, not being permanently institutionalized, being aware of the diagnosis, not being terminally ill (a life expectancy of more than six months according to their general practitioner), being mentally capable of participating, and having sufficient mastery of the Dutch language. Every year 500 new panel members are selected to replace panel members who have withdrawn or who have participated for the maximum term of four years. The NPCD can be considered to be representative of the chronic disease population in the Netherlands of aged 15 years and older. The NPCD is registered with the Dutch Data Protection Authority. All data are collected and handled in accordance with the privacy protection guidelines of the Dutch authority.

Patients voluntarily participate in the NPCD. Participation has no influence on their care. Twice a year (spring and autumn) the panel members voluntarily fill out a questionnaire. The panel members could choose whether they wanted to receive questionnaires by post, email or phone. Some items used in this study were issued in the spring questionnaire of 2014, others in the autumn questionnaire of 2014. The questionnaire can be found in Additional file 1.

In addition, to test our hypothesis regarding self-monitoring of health data by people with different chronic disease types an expert panel of 16 medical doctors and physiotherapists was invited to participate in a questionnaire study in February and March 2016.

### Measurements

#### Participant characteristics

The background characteristics of the members of the NPCD had already been gathered using a questionnaire that was completed at inclusion in the panel. For this study, the following characteristics were used: gender (1 = male, 2 = female), age and level of education (1 = low (primary school or preparatory vocational training), 2 = middle (intermediate or advanced general education or intermediate vocational training), 3 = high (high vocational education or university)). In addition, information regarding participants’ chronic disease(s) was provided at inclusion by their general practitioner.

#### Self-monitoring of health data

The following question regarding self-monitoring of health data was asked to participants in autumn 2014: “Did you measure certain health data by yourself in the past year, for example blood pressure, blood glucose values or lung function?” Participants could answer: 1) yes; 2), no, but I would like to do this (independently); 3), no, but I would like to do this together with a care professional; or 4), no, and I do not want to do this.

#### Self-efficacy – patient specific

Patients’ perceived self-efficacy was collected using the Dutch version of the general self-efficacy scale [21] in spring 2014 (Cronbach’s alpha was 0.92). This questionnaire consists of ten questions with a four-point Likert scale ranging from 1) completely wrong to 4) completely right. For example: “When I am confronted with a problem, I can usually find several solutions” and “It is easy for me to stick to my aims and accomplish my goals”. Participants with four or more missing values were excluded. Mean values were used in further analyses, in which a higher mean score indicates a higher level of self-efficacy.

#### Problems in daily functioning – patient specific

The Dutch version of the Physical Health Composite Score (PCS) of the SF-12 [22] was used to investigate patients’ experienced problems in daily functioning in autumn 2014 (Cronbach’s alpha was 0.88). The SF-12 has shown adequate validity and reliability in multiple studies [23]. PCS scores were collected in spring 2014. Mean scores were calculated using QualityMetric Health Outcomes™ Scoring Software 5.0 and could range from 0 to 100, in which 100 indicates the highest level of health.

#### Disease control – disease specific

To test the hypothesis, based on literature and experiences regarding self-monitoring of health data by people with different disease types (disease-specific hypothesis), nine care professionals from the expert panel (six medical doctors and three physiotherapists) out of sixteen experts who were invited, answered the following question for 17 different chronic diseases: “To what extent can people with a chronic disease, in general, independently keep their disease under control (by means of nutrition, physical activity, medication etc.)?” Participants could respond with: 1) not at all; 2) to some extent; or 3) to a large extent. Mean scores per disease type were used in the analyses.

### Statistical analyses

Descriptive analyses were conducted to study participants’ characteristics per disease group. Participants were divided into 17 different disease-type categories based on the diagnosis of their first chronic disease (ischaemic heart disease, hypertension, other cardiovascular disorders, cancer, asthma, COPD, other respiratory diseases, diabetes, thyroid disorder, chronic back pain, rheumatism, osteoarthritis, other musculoskeletal disorders, migraine, other neurological disorders, digestive disorder and skin disease). The most common diseases per disease category can be found in Additional file 2.

Univariate logistic regression analyses were conducted to test the relationship between patient- and disease-specific characteristics, and patients’ willingness to self-monitor health data (dependent variable: 1 = participants who did measure certain health data by themselves + participants who would like to do that (independently), 0 = participants who would like to do that together with a care professional + participants who did not want to do that at all). The univariate logistic regression analyses were conducted with the following independent variables: mean scores of the expert panel regarding disease controllability, mean score of the general self-efficacy scale, PCS score of the SF-12 and age, gender (1 = male, 2 = female), level of education (1 = low, 2 = middle, 3 = high) and multimorbidity (0 = one disease, 1 = two or more diseases). Assumptions for logistic regression were checked. We adjusted for clustering of data within chronic disease types (patients within one disease group have the same disease control score). Finally, multivariate logistic regression analyses were performed with all the above-mentioned concepts (dependent variable: willingness to self-monitor). Statistical analyses were performed using STATA 14.0.

## Results

### Participants

**Fig. 1:**
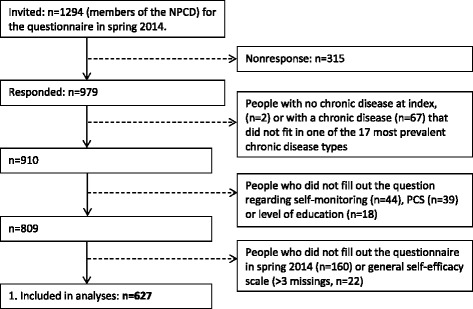
Flow chart of participants included in the study

Figure 1 shows a flow chart of the process of the inclusion of participants in this study. Out of 1294 participants of the NPCD, 979 responded to the questionnaire that was issued in spring 2014. Of these 979 participants, 2 had no chronic disease or a disease that did not fit in one of the 17 most prevalent chronic disease types (*n* = 67). Subsequently 101 participants were excluded because of incomplete data regarding the self-monitoring question (*n* = 44), PCS (*n* = 39) and level of education (*n* = 18). In addition 160 participants were excluded because they did not fill out the questionnaire at spring (*n* = 160) or because of incomplete data in the general self-efficacy scale (*n* = 22). This resulted in a total sample of 627 participants. Non response analyses showed no differences in characteristics between the non-responders (including people who responded but did not fill out the entire questionnaire) and the final sample, except for age (non-responders: M = 63.5, SE = 0.55; final sample: M = 65.1, SE = 0.46, *t*(1292) = −2.15, *p* = 0.03). So except for a sampling bias of age the sample is representative of the chronic disease population in the Netherlands.

Table 1 shows the characteristics of the study sample. Diabetes (19.8%), other cardiovascular disorders (8.9%), ischaemic heart disease/heart failure (8.6%) and asthma (8.3%) were the most common chronic disease types within the sample. Almost 4% of the participants were diagnosed less than three years ago, 16.1% three to five years ago, 31.9% six to ten years ago and almost half (48.1%) longer than ten years ago. More than half (51.2%) had been diagnosed with two or more chronic diseases. All characteristics per disease group can be found in Additional file 3.

**Table 1:** Characteristics of the study sample

Characteristics		Study sample (*n* = 627) Mean (sd) or n (%)	Non response (*n* = 667) Mean (sd) or n (%)
Age in years		65.1 (sd = 11.6)	63.5 (sd = 14.3)
Gender	Male	313 (49.9%)	313 (46.9%)
Level of education	Low	199 (31.7%)	220 (32.98%)
	Medium	276 (44.0%)	276 (41.38%)
	High	152 (24.2%)	122 (18.29%) Missing = 49 (7.4%)
Chronic condition	One	306 (48.8%)	321 (48.1%)
	Two or more	321 (51.2%)	343 (51.4%) Missing = 3 (0.5%)
Data collection	By post	376 (60.9%)	
	Online	248 (39.6%)	
	By telephone	3 (0.5%)	
General self-efficacy		3.12 (sd = 0.6)	
Physical Health Composite Score		42.81 (sd = 11.4)	
Willing to self-monitor		348 (55.5%)	

### Relationship of disease controllability with willingness to self-monitor

**Fig. 2:**
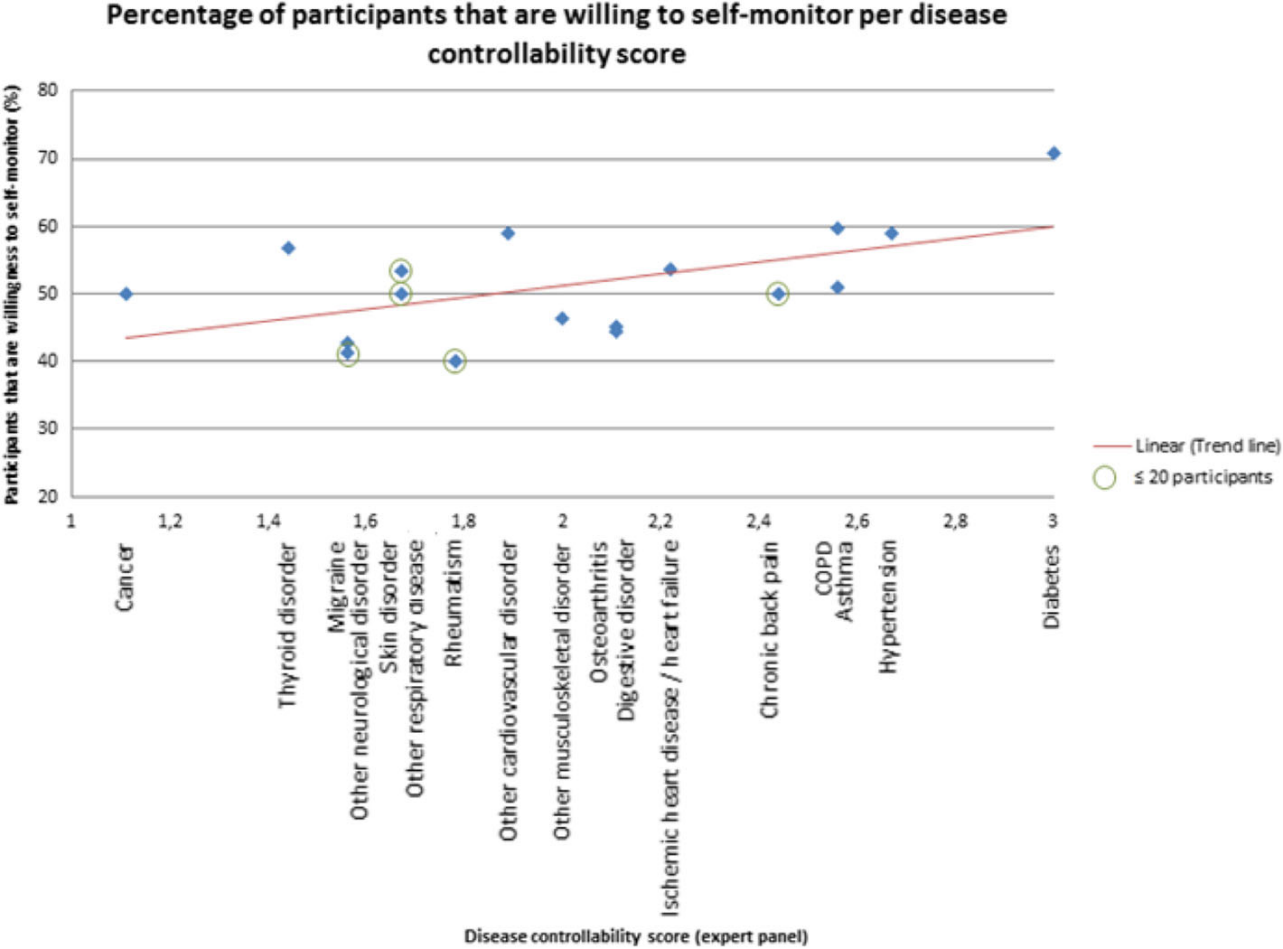
Mean disease controllability score (expert panel: 1 = not at all; 2 = to some extent; 3 = to a large extent) plotted against the percentage of participants that is willing to monitor independently

Figure 2 represents the association between disease controllability scores (assessed by the expert panel) and the percentage of participants that is willing to self-monitor health data per disease type. Patients’ willingness to self-monitor differs greatly among disease types: patients with diabetes (71.0%), asthma (59.6%) and hypertension (59.1%) were most willing to self-monitor. In contrast, patients with rheumatism (40.0%), migraine (41.2%) and other neurological disorders (42.9%) were less willing to self-monitor. In addition, the expert panel assessed diabetes (3.0), hypertension (2.7) and COPD and asthma (2.6) as diseases that can be kept well under control by the patient, and cancer (1.1), thyroid disorder (1.4), and other neurological disorders and migraine (1.6) as the diseases that are most difficult for the patient to keep under control. The scores of the expert panel can be found in Additional file 4.

A relationship is found between disease controllability scores and patients’ willingness to self-monitor. The correlation between disease controllability scores and the percentage of participants that is willing to self-monitor is significant (*r* = 0.547, *p* < 0.05). In addition, looking at the univariate and multivariate logistic association of disease controllability with patients’ willingness to self-monitor (first column Tables 2 and 3 respectively), a significant association is found (univariate: OR = 1.589, 95% CI = 1.142–2.210; multivariate: OR = 1.639, 95% CI = 1.129–2.380).

**Table 2:** Univariate logistic regression analyses with the dependent variable: willingness to monitor health data

	Entire sample (*n* = 627)		Sample without diabetes (*n* = 503)		Diabetes only (*n* = 124)	
Independent variable	*OR (95% CI)*	*p*	*OR (95% CI)*	*p*	*OR (95% CI)*	*p*
Age	0.994 (0.979–1.010)	0.453	0.994 (0.977–1.012)	0.519	0.973 (0.934-1.014)	0.193
Gender (ref = male)	0.728 (0.533–0.993)	*0.045*	0.669 (0.488–0.916)	*0.012*	1.800 (0.774–4.187)	0.172
Level of education (ref = low)	–	–	–	–	–	–
Intermediate	1.308 (0.863–1.983)	0.206	1.611 (1.158–2.240)	*0.005*	0.741 (0.308–1.784)	0.503
High	1.908 (1.300–2.801)	*0.001*	2.254 (1.603–3.170)	*<0.001*	1.256 (0.435-3.627)	0.673
Multimorbidity (ref = one disease)	1.076 (0.816–1.420)	0.604	1.192 (0.902–1.577)	0.217	0.849 (0.390–1.850)	0.681
Disease control score (expert panel)	1.589 (1.142–2.210)	*0.006*	1.169 (0.942–1.451)	0.156	–	–
Physical Health Composite Score (PCS)	1.005 (0.989–1.020)	0.567	1.002 (0.984–1.020)	0.823	1.003 (0.966–1.041)	0.874
Self-efficacy patient	0.994 (0.747–1.322)	0.968	1.062 (0.760–1.483)	0.724	0.747 (0.374–1.492)	0.408

**Table 3:** Multivariate logistic regression analyses with the dependent variable: willingness to monitor health data

	Entire sample (*n* = 627)		Sample without diabetes (*n* = 503)		Diabetes only (*n* = 124)	
Independent variable	*OR (95% CI)*	*p*	*OR (95% CI)*	*p*	*OR (95% CI)*	*p*
Age	0.992 (0.976–1.008)	0.312	0.993 (0.974–1.012)	0.468	0.972 (0.929–1.018)	0.229
Gender (ref = male)	0.792 (0.522–1.203)	0.274	0.655 (0.468–0.916)	*0.013*	1.934 (0.783–4.776)	0.153
Level of education (ref = low)	–	–	–	–	–	–
Intermediate	1.491 (0.973–2.284)	0.067	1.804 (1.256–2.590)	*0.001*	0.652 (0.250–1.701)	0.382
High	2.042 (1.415–2.950)	*<0.001*	2.344 (1.645–3.341)	*<0.001*	1.217 (0.398–3.724)	0.730
Multimorbidity (ref = one disease)	1.170 (0.868–1.576)	0.303	1.278 (0.903–1.809)	0.167	0.876 (0.386–1.988)	0.751
Disease control score (expert panel)	1.639 (1.129–2.380)	*0.009*	1.164 (0.908–1.491)	0.230	–	–
Physical Health Composite Score (PCS)	1.001 (0.984–1.019)	0.916	0.999 (0.979–1.020)	0.934	1.008 (0.962–1.057)	0.726
Self-efficacy patient	0.862 (0.655–1.134)	0.288	0.881 (0.636–1.221)	0.448	0.757 (0.346–1.656)	0.486

### Diabetes group – disease controllability and willingness to self-monitor

As can be seen in Fig. 2, all experts assessed diabetes as a disease that can, to a large extent, be kept under control by the patient (the maximum mean score of 3.0). In addition, the diabetes group scored remarkably high on willingness to self-monitor (71.0%). Therefore, we decided to look more deeply into the diabetes group only (*n* = 124).

Of the 124 people with diabetes, 9 participants have type I diabetes and 103 type II (for 12 participants it is unknown what type of diabetes they have). 41 participants with diabetes use insulin (33.1%), 65 do not use insulin (52.4%) and for 18 participants this is unknown (14.5%). Of the 41 participants using insulin, 95.1% are willing to self-monitor health data. In contrast, among patients who are not using insulin 46.2% are willing to do so.

In Tables 2 and 3 (second column) it can be seen that by excluding the entire diabetes group in the univariate and multivariate logistic regression analyses to investigate the relationship between disease controllability and patients’ willingness to self-monitor (*n* = 503), no significant association is found (univariate: OR = 1.169, 95% CI = 0.942–1.451; multivariate: OR = 1.164, 95% CI = 0.908–1.491).

### Relationship of patient characteristics with willingness to self-monitor

Patients’ perceived problems in daily functioning (PCS) and self-efficacy have no significant association with their willingness to self-monitor (see Tables 2 and 3). Age and multimorbidity also have no relationship with willingness to self-monitor. In contrast, males and more highly educated people are significantly more willing to self-monitor their health data.

Looking at the diabetes sample only (Tables 2 and 3 third column), there was no significant association between gender and education level, and patients’ willingness to self-monitor.

## Discussion

### Principal results

This study provides the first evidence of an association between disease controllability and patients’ willingness to self-monitor health data. Against our expectations, no evidence is found for a relationship between self-efficacy and the severity of problems that patients experience with daily functioning, and patients’ willingness to self-monitor. In addition, it is found that males and more highly educated people are more willing to self-monitor their health data.

The scores of the diabetes group regarding disease controllability and patients’ willingness to self-monitor were remarkably high. Patients with diabetes using insulin were particularly willing to self-monitor (95.1%). The difference between diabetes and other chronic disease types regarding self-monitoring could be explained by the fact that for diabetes patients self-monitoring is recommended as an integral component of their treatment (particularly for patients using insulin) [24, 25]. So for many persons with diabetes, their “willingness” to self-monitor is beyond question, because they have to monitor their blood glucose level for their (optimal) treatment. Hence, as we found in this study, this is also independent of the patient characteristics they have. For other chronic disease types self-monitoring is often not yet integrated into the standard treatment. For these chronic disease types males and more highly educated people were more willing to self-monitor, which is in line with some, but not all, self-management research [26–28]. Interestingly, we did not find a relationship between multimorbidity (having two or more chronic conditions) and willingness to self-monitor. Although many research found that performing optimal self-management behaviour may be more challenging for people with multiple chronic diseases [29, 30], this study suggest that this does not influence willingness to self-monitor.

Contrary to our expectations no effect of self-efficacy on patients’ willingness to self-monitor was found in this study. However, in other studies that did find an association between self-efficacy and self-monitoring, questionnaires were used to investigate self-efficacy regarding patients’ chronic disease, such as how participants assessed their capability to monitor, plan and carry out activities for their disease (for example nutrition, physical exercises and medication) [16, 17]. In the current study the general self-efficacy scale [21] was used, which consists of generic questions such as “When I am confronted with a problem, I can usually find several solutions” and “It is easy for me to stick to my aims and accomplish my goals”. It appears that patients’ general self-efficacy is not related to their willingness to self-monitor. In future research it might be interesting to investigate if a chronic disease self-efficacy scale, e.g., the Chronic Diseases Self-Efficacy Scale of Lorig and colleagues [31] is related to willingness to self-monitor.

No relationship was found between patients’ perceived problems in daily functioning and self-monitoring, which is not in line with our hypothesis. Again, participants were asked to assess all their general health problems in daily functioning, and not only the physical problems related to their specific chronic disease. Although we found some support for this possible relationship in our recently performed focus group study [13], other research regarding eHealth did not find a relationship between health needs and patients’ acceptance and interests of eHealth as well [32, 33]. It seems that patients’ willingness to self-monitor health data is not directly related to their perceived health problems. In future research it might be interesting to investigate if a disease specific health scale (such as Quality of life Disease Impact Scale [34]) has an influence on patients’ willingness to self-monitor. In addition, although we did not ask participants to indicate their expected or perceived benefits of self-monitoring health data, it might be expected that willingness to self-monitor is more related to the overall concepts of “perceived benefits” and “perceived usefulness”, which are well-studied concepts in care technology acceptance research [8, 19, 35–37]. It might be that patients are more willing to self-monitor when they believe that self-monitoring can convey (health) benefits.

This study provides the first evidence that patients’ willingness to self-monitor might be associated with disease controllability. Further research should investigate this association more deeply and should focus on how disease controllability influences willingness to self-monitor. In the current study disease controllability is investigated using one general question in an expert panel (“To what extent can people with a chronic disease, in general, independently keep their disease under control (by means of nutrition, physical activity, medication etc.) for the following chronic diseases?”). It is recommended to first define the concept of disease controllability and to investigate what factors and mechanisms play a role in this. Secondly, it should be investigated how disease controllability influences patients’ willingness to self-monitor, for example by using qualitative methodology focusing on behavioural and motivational aspects of patients. Thirdly, it should be investigated how self-monitoring applications for different disease types can be adapted to improve this. In addition, it should be investigated what other disease- and patient specific factors play a role in patients’ willingness to self-monitor, such as disease effects, patients’ perceived controllability of symptoms and patients’ coping and attitudes toward their disease. This study shows that the percentage of participants that is willing to self-monitor health data differed greatly between disease types. Hence, while developing and offering self-monitoring applications it should be kept in mind that not all patient groups are willing to self-monitor their health data.

### Strengths and limitations

The strength of this study is its general focus on patients’ willingness to self-monitor health data in a broad sample (*n* = 627) of people with the most common chronic somatic disease types (17 chronic disease types). The panel used for this study was representative of the Dutch chronic disease population (except for age). The overall response of this panel is high and participants were not recruited for the specific topic of this study which minimizes selection bias; items used for this study were a part of a panel questionnaire. In addition, this study is conducted to test our hypotheses, which were based on the results of a recently performed focus group study [13]. Moreover, to the best of our knowledge, this is the first study to investigate the relationship between the controllability of certain chronic disease types and patients’ willingness to self-monitor.

As mentioned before, one limitation of this study is that data from general questionnaires have been used to investigate patients’ self-efficacy and physical problems that were not specifically related to patients’ chronic disease. In addition, the dependent variable ‘patients’ willingness to self-monitor’ was based on one non-validated question. Furthermore, patients’ willingness to self-monitor and their Physical health Composite Scores (PCS) were collected in autumn 2014, in contrast to general self-efficacy scores, which were investigated in spring 2014. However, it is expected that these scores did not significantly change within six months. In addition, the number of people within a disease type highly differed from *n* = 6 (chronic back pain) to *n* = 124 (diabetes). Five chronic disease types had only 20 or less participants. In addition, in the analyses with the diabetes group only, no relationships were found between gender and education level, and patients’ willingness to self-monitor. This might be explained by the lower number of participants compared with the entire sample (*n* = 124 vs *n* = 627). Moreover, no relationship between multimorbidity and patients’ willingness to self-monitor was found in this study. We defined multimorbidity as having two or more chronic conditions. It might be that this does not reflect the complexity of this problem, in particular not for people with a high number of conditions.

Another limitation is that people that had done self-monitoring in the previous year and those that wanted to do so independently were recoded as being willing to self-monitor. Additional separate analyses were performed to investigate differences between the association of the actual self-monitoring group or the willing to self-monitor group on the one hand, and disease controllability on the other. Although similar positive associations between the actual and willing to self-monitor group, and disease controllability were found, no valid statements could be made due to the small number of people in each (disease) group. In addition, it is assumed that the actual self-monitoring group were also willing to self-monitor in the first place. Moreover, participants that wanted to do self-monitoring with the help of a care professional and participants that did not want to do self-monitoring at all were recoded as being not willing to self-monitor, because we were particularly interested in people that were willing to self-monitor independently. This because we consider self-monitoring as a core element of self-management and by monitoring independently the required effects of self-monitoring (improving symptom management, disease regulation, patients’ coping and attitudes toward their disease, realistic goal setting and an enhanced quality of life [6]) will be most effective.

## Conclusion

This study provides the first evidence that patients’ willingness to self-monitor might be associated with disease controllability. Further research should investigate this association more deeply and should focus on how disease controllability influences willingness to self-monitor. In addition, it should be investigated what other disease- and patient specific factors play a role in patients’ willingness to self-monitor. No evidence is found of a relationship between self-efficacy and the severity of problems that patients experience with daily functioning, and patients’ willingness to self-monitor. Since the percentage of participants that is willing to self-monitor health data differed greatly between disease types, it should be taken into account that not all patient groups are willing to self-monitor their health data.

## Additional files

Additional file 1: Questionnaire NPCD: Items of the questionnaire for NPCD panel members. (DOCX 15 kb)
Additional file 2: Chronic diseases: Most common chronic diseases per disease category. (DOCX 16 kb)
Additional file 3: Patient characteristics: Patients’ characteristics per disease type. (DOCX 19 kb)
Additional file 4: Results expert panel: Results of the expert panel of disease controllability per disease category. (DOCX 17 kb)

## Abbreviations

COPD: Chronic obstructive pulmonary disease
NPCD: National panel of people with chronic illness or disability
PCS: Physical health composite score
TAM: Technology acceptance model
