# Supplementary material for: Self-monitoring of health data by patients with a chronic disease: does disease controllability matter?
Source: BMC Fam Pract. 2017 Mar 20;18:40. doi: 10.1186/s12875-017-0615-3 (PMC5360032; doi:10.1186/s12875-017-0615-3)
Supplement: Additional file 1: — Questionnaire NPCD: Items of the questionnaire for NPCD panel members. (DOCX 15 kb) [file 12875_2017_615_MOESM1_ESM.docx]

*Additional file 1. Items of the questionnaire for NPCD panel members.*

**Question regarding self-monitoring**1. Did you measure certain health data by yourself in the past year, for example blood pressure, blood glucose values or lung function?
*Response format: 1=yes, 2=no, but I would like to do this (independently), 3=no, but I would like to do this together with a care professional, 4=no, and I do not want to do* this.

**General self-efficacy scale**1. I can always manage to solve difficult problems if I try hard enough.
2. If someone opposes me, I can find the means and ways to get what I want.
3. It is easy for me to stick to my aims and accomplish my goals.
4. I am confident that I could deal efficiently with unexpected events.
5. Thanks to my resourcefulness, I know how to handle unforeseen situations.
6. I can solve most problems if I invest the necessary effort.
7. I can remain calm when facing difficulties because I can rely on my coping abilities.
8. When I am confronted with a problem, I can usually find several solutions.
9. If I am in trouble, I can usually think of a solution.
10. I can usually handle whatever comes my way.
*Response Format: 1=Not at all true, 2=Hardly true, 3=Moderately true, 4=Exactly true*

**SF-12 health survey**1. In general, how would you call your health?
*Response Format: 1=Excellent, 2=Very good, 3=Good, 4=Fair, 5= Poor*
- The following questions are about activities you might do on a typical day. Does your health limit you a lot, a little or not at all during these activities?
*Response Format: 1=Yes, limited a lot, 2=Yes, limited a little, 3=No, not limited at all*
2. In moderate activities, such as moving a table, pushing a vacuum cleaner, cycling?
3. How about climbing several flights of stairs?
4. During the past four weeks, have you accomplished less than you would like as a result of your physical health?
*Response Format: 1=Yes, 2=No*
5. During the past four weeks, were you limited in the kind of work or other activities you could do as a result of your physical health?
*Response Format: 1=Yes, 2=No*
6. During the past four weeks, have you accomplished less than you would like as a result of your emotional health (such as feeling depressed or anxious).
*Response Format: 1=Yes, 2=No*
7. During the past four weeks, were you limited in the kind of work or other activities you could do as a result of your emotional health (such as feeling depressed or anxious).
*Response Format: 1=Yes, 2=No*
8. During the past four weeks, how much did pain interfere with your normal work, including both work outsight the home and housework).
*Response Format: 1=Yes, 2=No*
9. During the past four weeks, how often have you felt calm and peach full?
*Response Format: 1=all of the time, 2=most of the time, 3=a good bit of the time, 4=some of the time, 5=a little of the time, 6=none of the time.*
10. During the past four weeks, how often did you have a lot of energy?
*Response Format: 1=all of the time, 2=most of the time, 3=a good bit of the time, 4=some of the time, 5=a little of the time, 6=none of the time.*
11. During the past four weeks, how often have you felt downhearted or blue?
*Response Format: 1=all of the time, 2=most of the time, 3=a good bit of the time, 4=some of the time, 5=a little of the time, 6=none of the time.*
12. During the past four weeks, how much of the time has your physical health and emotional problems interfered with your social activities (like visiting friends and relatives)?
*Response Format: 1=all of the time, 2=most of the time, 3=a good bit of the time, 4=some of the time, 5=a little of the time, 6=none of the time.*
