# Supplementary material for: Self-monitoring of health data by patients with a chronic disease: does disease controllability matter?
Source: BMC Fam Pract. 2017 Mar 20;18:40. doi: 10.1186/s12875-017-0615-3 (PMC5360032; doi:10.1186/s12875-017-0615-3)
Supplement: Additional file 2: — Chronic diseases: Most common chronic diseases per disease category. (DOCX 16 kb) [file 12875_2017_615_MOESM2_ESM.docx]

*Additional file 2. Most common chronic diseases per disease category.*

| **Disease category** | **ICPC** | **Name** |
| --- | --- | --- |
| Ischemic heart disease | K74 | Ischaemic heart disease w. angina |
|  | K75 | Acute myocardial infarction |
|  | K76 | Ischaemic heart disease w/o angina |
|  | K77 | Heart failure |
| Hypertension | K86 | Hypertension uncomplicated |
|  | K87 | Hypertension complicated |
| Other cardiovascular disorder | K78 | Atrial fibrillation/flutter |
|  | K84 | Heart disease other |
|  | K90 | Stroke/cerebrovascular accident |
|  | K92 | Atherosclerosis/PVD |
| Cancer | X76 | Malignant neoplasm breast female |
|  | X77 | Malignant neoplasm genital other (f) |
|  | D75 | Malignant neoplasm colon/rectum |
|  | Y77 | Malignant neoplasm prostate |
|  | U76 | Malignant neoplasm of bladder |
| Asthma | R96 | Asthma |
| COPD | R95 | Chronic obstructive pulmonary dis |
|  | R91 | Chronic bronchitis/bronchiectasis |
| Other respiratory disease | R97 | Allergic rhinitis |
| Diabetes | T90 | Diabetes |
| Thyroid disorder | T85 | Hyperthyroidism/thyrotoxicosis |
|  | T86 | Hypothyroidism/myxoedema |
| Chronic back pain | L02 | Back symptom/complaint |
|  | L03 | Low back symptom/complaint |
|  | L86 | Back syndrome with radiating pain |
| Rheumatism | L88 | Rheumatoid/seropositive arthritis |
| Osteoarthritis | L90 | Osteoarthrosis of knee |
|  | L91 | Osteoarthrosis other |
| Other musculoskeletal disorder | L95 | Osteoporosis |
|  | L99 | Musculoskeletal disease, other |
| Migraine | N89 | Migraine |
| Other neurological disorder | N86 | Multiple sclerosis |
|  | N88 | Epilepsy |
|  | N99 | Neurological disease, other |
| Digestive disorder | D92 | Diverticular disease |
|  | D93 | Irritable bowel syndrome |
|  | D94 | Chronic enteritis/ulcerative colitis |
| Skin disease | S91 | Psoriasis |
|  | S87 | Dermatitis/atopic eczema |
