# Supplementary material for: Self-monitoring of health data by patients with a chronic disease: does disease controllability matter?
Source: BMC Fam Pract. 2017 Mar 20;18:40. doi: 10.1186/s12875-017-0615-3 (PMC5360032; doi:10.1186/s12875-017-0615-3)
Supplement: Additional file 3: — Patient characteristics: Patients’ characteristics per disease type. (DOCX 19 kb) [file 12875_2017_615_MOESM3_ESM.docx]

*Additional file 3. Patients’ characteristics per disease type*

| **Disease** | **Number**  n, (%) | **Age**  mean, (sd) | **Gender (male)**  n, (%) | **Level of education** | | | **Multi-morbi-**  **dity**  **(1 disease)**  n, (%) | **PCS**  mean, (sd) | **Self-efficacy**  mean, (sd) |
| --- | --- | --- | --- | --- | --- | --- | --- | --- | --- |
|  |  |  |  | ***Low***  n, (%) | ***Interme-***  ***diate***  n, (%) | ***High***  n, (%) |  |  |  |
| Ischemic heart disease | 54  (8.6%) | 70.4  (sd=10.1) | 45  (83.3%) | 23  (42.6%) | 15  (27.8%) | 16  (29.6%) | 22  (40.7%) | 44.1  (sd=10.6) | 3.1  (sd=0.5) |
| Hypertension | 22  (3.5%) | 68.9,  (sd= 11.6) | 13  (59.1%) | 10  (45.5%) | 9  (40.9%) | 3  (13.6%) | 9  (40.9%) | 43.8  (sd=11.0) | 3.2  (sd=0.6) |
| Other cardiovascular disorder | 56  (8.9%) | 69.3,  (sd= 8.8) | 34  (60.7%) | 21  (37.5%) | 23  (41.1%) | 12  (21.4%) | 26  (46.4%) | 41.3  (sd=9.9) | 3.1  (sd=0.5) |
| Cancer | 46  (7.3%) | 65.2,  (sd= 10.4) | 14  (30.4%) | 10  (21.7%) | 25  (54.4%) | 11  (23.9%) | 23  (50.0%) | 43.1  (sd=13.4) | 3.3  (sd=0.6) |
| Asthma | 52  (8.3%) | 61.2,  (sd= 13.1) | 24  (46.2%) | 9  (17.3%) | 26  (50.0%) | 17  (32.7%) | 26  (50.0%) | 41.9  (sd=11.7) | 3.3  (sd=0.6) |
| COPD | 47  (7.5%) | 67.2,  (sd= 10.7) | 26  (55.3%) | 20  (42.6%) | 19  (40.4%) | 8  (17.0%) | 24  (51.1%) | 40.6  (sd=12.4) | 3.1  (sd=0.5) |
| Other respiratory disease | 15  (2.4%) | 54.5,  (sd= 12.1) | 5  (33.3%) | 1  (6.7%) | 7  (46.7%) | 7  (46.7%) | 8  (53.3%) | 44.5  (sd=12.6) | 3.1  (sd=0.8) |
| Diabetes | 124  (19.8%) | 66.9,  (sd= 9.9) | 78  (62.9%) | 47  (37.9%) | 47  (37.9%) | 30  (24.2%) | 69  (55.7%) | 44.8  (sd=10.4) | 3.1  (sd=0.6) |
| Thyroid disorder | 37  (5.9%) | 63.4,  (sd= 12.8) | 5  (13.5%) | 9  (24.3%) | 23  (62.2%) | 5  (13.5%) | 19  (51.4%) | 43.3  (sd=9.3) | 3.1  (sd=0.5) |
| Chronic back pain | 6  (1.0%) | 59.2,  (sd= 4.6) | 5  (83.3%) | 3  (50.0%) | 2  (33.3%) | 1  (16.7%) | 2  (33.3%) | 35.0  (sd=10.8) | 2.7  (sd=0.4) |
| Rheumatism | 15  (2.4%) | 60.8,  (sd= 10.1) | 6  (40.0%) | 4  (26.7%) | 9  (60.0%) | 2  (13.3%) | 8  (53.3%) | 35.6  (sd=9.3) | 3.1  (sd=0.5) |
| Osteoarthritis | 31  (4.9%) | 65.2,  (sd= 9.4) | 11  (35.5%) | 15  (48.4%) | 11  (35.5%) | 5  (16.1%) | 13  (41.9%) | 36.0  (sd=10.7) | 2.9  (sd=0.7) |
| Other musculoskeletal disorder | 28  (4.5%) | 67.8,  (sd= 12.9) | 4  (14.3%) | 5  (17.9%) | 16  (57.1%) | 7  (25.0%) | 9  (32.1%) | 40.1  (sd=12.9) | 3.1  (sd=0.6) |
| Migraine | 17  (2.7%) | 55.4,  (sd= 10.3) | 4  (23.5%) | 3  (17.7%) | 10  (58.8%) | 4  (23.5%) | 10  (58.8%) | 45.4  (sd=14.1) | 2.9  (sd=0.5) |
| Other neurological disorder | 21  (3.4%) | 62.1,  (sd= 10.3) | 14  (66.7%) | 5  (23.8%) | 7  (33.3%) | 9  (57.1%) | 11  (52.4%) | 43.5  (sd=12.7) | 3.3  (sd=0.5) |
| Digestive disorder | 36  (5.7%) | 64.0,  (sd= 12.0) | 14  (38.9%) | 9  (25.0%) | 20  (55.6%) | 7  (19.4%) | 16  (44.4%) | 47.7  (sd=10.2) | 3.2  (sd=0.5) |
| Skin disease | 20  (3.2%) | 54.2,  (sd= 14.5) | 11  (55.0%) | 5  (25.0%) | 7  (35.0%) | 8  (40.0%) | 11  (55.0%) | 45.6  (sd=8.3) | 3.1  (sd=0.5) |
