# Supplementary material for: Self-monitoring of health data by patients with a chronic disease: does disease controllability matter?
Source: BMC Fam Pract. 2017 Mar 20;18:40. doi: 10.1186/s12875-017-0615-3 (PMC5360032; doi:10.1186/s12875-017-0615-3)
Supplement: Additional file 4: — Results expert panel: Results of the expert panel of disease controllability per disease category. (DOCX 17 kb) [file 12875_2017_615_MOESM4_ESM.docx]

*Additional file 4. Results of the expert panel of disease controllability per disease category.*

To what extent can people with a chronic disease, in general, independently keep their disease under control (by means of nutrition, physical activity, medication etc.)?

1. Number of experts per score

| **Disease type** | **not at all** | **to some extent** | **to a large extent** |
| --- | --- | --- | --- |
| Cancer | 8 | 1 |  |
| Thyroid disorder | 5 | 4 |  |
| Other neurological disorder | 4 | 5 |  |
| Migraine | 4 | 5 |  |
| Skin disorder | 4 | 4 | 1 |
| Other respiratory disease | 4 | 4 | 1 |
| Rheumatism | 2 | 7 |  |
| Other cardiovascular disorder | 2 | 6 | 1 |
| Other musculoskeletal disorder | 2 | 5 | 2 |
| Osteoarthritis | 2 | 4 | 3 |
| Digestive disorder | 2 | 4 | 3 |
| Ischemic heart disease / heart failure |  | 7 | 2 |
| Chronic back pain | 1 | 3 | 5 |
| COPD |  | 4 | 5 |
| Asthma | 1 | 2 | 6 |
| Hypertension |  | 3 | 6 |
| Diabetes |  |  | 9 |

2. Calculated mean scores and standard deviation

| **Disease type** | **not at all**  **(1)** | **to some extent (2)** | **to a large extent (3)** | **Mean score** | **Sd** |
| --- | --- | --- | --- | --- | --- |
| Cancer | 8 | 2 |  | 1.11 | .33 |
| Thyroid disorder | 5 | 8 |  | 1.44 | .53 |
| Other neurological disorder | 4 | 10 |  | 1.56 | .53 |
| Migraine | 4 | 10 |  | 1.56 | .53 |
| Skin disorder | 4 | 8 | 3 | 1.67 | .71 |
| Other respiratory disease | 4 | 8 | 3 | 1.67 | .71 |
| Rheumatism | 2 | 14 |  | 1.78 | .44 |
| Other cardiovascular disorder | 2 | 12 | 3 | 1.89 | .60 |
| Other musculoskeletal disorder | 2 | 10 | 6 | 2.00 | .71 |
| Osteoarthritis | 2 | 8 | 9 | 2.11 | .78 |
| Digestive disorder | 2 | 8 | 9 | 2.11 | .78 |
| Ischemic heart disease / heart failure |  | 14 | 6 | 2.22 | .44 |
| Chronic back pain | 1 | 6 | 15 | 2.44 | .73 |
| COPD |  | 8 | 15 | 2.56 | .53 |
| Asthma | 1 | 4 | 18 | 2.56 | .73 |
| Hypertension |  | 6 | 18 | 2.67 | .50 |
| Diabetes |  |  | 27 | 3.00 | 0 |
